# The “Sniffin' Kids” Test - A 14-Item Odor Identification Test for Children

**DOI:** 10.1371/journal.pone.0101086

**Published:** 2014-06-30

**Authors:** Valentin A. Schriever, Eri Mori, Wenke Petters, Carolin Boerner, Martin Smitka, Thomas Hummel

**Affiliations:** 1 Smell & Taste Clinic, Department of Otorhinolaryngology, University Hospital Carl Gustav Carus, Technische Universität (TU) Dresden, Dresden, Germany; 2 Department of Neuropediatrics, University Hospital Carl Gustav Carus, Technische Universität (TU) Dresden, Dresden, Germany; 3 Department of Otorhinolaryngology, Jikei University, School of Medicine, Tokyo, Japan; Center for Genomic Regulation, Spain

## Abstract

Tools for measuring olfactory function in adults have been well established. Although studies have shown that olfactory impairment in children may occur as a consequence of a number of diseases or head trauma, until today no consensus on how to evaluate the sense of smell in children exists in Europe. Aim of the study was to develop a modified “Sniffin' Sticks” odor identification test, the “Sniffin' Kids” test for the use in children. In this study 537 children between 6-17 years of age were included. Fourteen odors, which were identified at a high rate by children, were selected from the “Sniffin' Sticks” 16-item odor identification test. Normative date for the 14-item “Sniffin' Kids” odor identification test was obtained. The test was validated by including a group of congenital anosmic children. Results show that the “Sniffin' Kids” test is able to discriminate between normosmia and anosmia with a cutoff value of >7 points on the odor identification test. In addition the test-retest reliability was investigated in a group of 31 healthy children and shown to be ρ = 0.44. With the 14-item odor identification “Sniffin' Kids” test we present a valid and reliable test for measuring olfactory function in children between ages 6–17 years.

## Introduction

The evaluation of the chemical senses has gained more interest in recent years. The administration of smell tests is widely used in clinical routine, especially in ENT and neurological clinics [1]. Several tests have been established as instruments for measuring olfactory function. In Northern America the University of Pennsylvania Smell Identification Test (UPSIT) is broadly used [2], while in Europe the administration of the “Sniffin' Sticks” test battery is used more commonly [3], [4]; in Japan the T&T olfactometer has been the standard for the last decades [5]. These tests were developed for distinguishing normosmia from hyposmia/anosmia in adults [2], [3], [6]. Despite the fact, that all tests have been used in children (e.g. [3], [7]), they are not well suited for children, due to the lengths of the test and possible unfamiliarity of the odors to young children. Therefore many clinics and laboratories used self-made olfactory tests when evaluating and studying olfactory function of children [9]–[13]. Most of these tests were not well evaluated and therefore the study results were difficult to compare to each other. Only in recent years the development of olfactory tests, especially designed for the administration in children, has been undertaken [14]–[18]. The “smell wheel” and the olfactory test of the NIH Toolbox are based on the UPSIT and the scratch and sniff technique [14], [16]. Both tests are for use in the USA or at least are aiming at English speaking children. Another odor identification test using squeeze bottles was developed in Australia [15]. None of these tests have gained wide distribution in Europe. A few studies have been conducted in Europe addressing this issue. The short version of the “Sniffin' Sticks” odor identification test was evaluated in Dutch children between the age of 6 and 11 years [8]. To our knowledge this test was not evaluated in other countries for children. In a recent study conducted in Poland, a short 6-item odor identification test was developed. So far this test is only used in Poland due to its odor selection and self-development it is commercially unavailable [18]. Since the “Sniffin' Sticks” 16-item odor identification test is commonly used for assessing olfactory function of adults in Europe, the primary aim of the current study was the evaluation of this test in a population between age 6–17 years. Secondly a modification of the 16-item odor identification test was planned to make it more applicable to children, which we named “Sniffin' Kids” Test, establishing a feasible method for odor identification testing for children.

## Material and Methods

### Ethics statement

For all study protocols the approval of the local Ethics Board of the Faculty of Medicine of the TU of Dresden had been obtained and all aspects of the study were performed in accordance to the Declaration of Helsinki. The study was explained to the parents and children in great detail, including the study design, procedure, tasks and possible risks. In addition to the verbal information given to the children/parents, written study information was provided separately for children and parents. Children under 8 years of age received verbal information only. Written informed consent was obtained from the parents. All participants gave their assent to participate in this study.

This study consisted of two parts. In part one the original “Sniffin' Sticks” 16-item odor identification test was applied to children between age 6 and 17 years. In the second part the odor identification test was modified according to the results from part one. This modified version was named “Sniffin' Kids” test.

### Part one

#### Participants

The data from 537 children, which underwent olfactory odor identification testing, was used for this study. The children were tested during the course of previously published [19], [20] or still ongoing studies. Children were recruited for each study using advertising flyers at the University Campus in Dresden, therefore representing the local population. In addition data was collected at the University of Dresden science fairs. For all children normal sense of smell was self-reported or reported by their parents by questionnaire (Do you have any problems with your sense of smell? Did you notice any problems with your child's sense of smell? Did he/she did not perceive an odor others were able to perceive?). None of the children suffered from any disease linked to olfactory dysfunction (e.g. diabetes mellitus, epilepsy, renal failure etc.). All children included in the study grew up in Germany and were fluent in the German language.

The mean age of the children was 11.9 years (SD 3.1, range 6–17) with a gender distribution of 268 girls and 269 boys (Table 1).

**Table 1:** Descriptive statistics of participants.

Group	Participants	Girls/Boys	Age (mean, SD, range)
**Control**	537	268/268	11.9, 3.1, 6–17
**Anosmic**	25	18/7	12, 2.7, 8–17

#### Testing

Testing took place in a quiet environment in a well-ventilated room. Each child was tested alone. All children were tested using the original “Sniffin' Sticks” 16-item odor identification test [21]. The use of the “Sniffin' Sticks” for odor presentation has been well evaluated in several studies [3], [4]. The “Sniffin' Sticks” are felt tip pens filled with odors. For odor presentation the cap is removed and each pen is presented approximately 2 cm under the nose for 3 seconds. The children were asked to identify the odors presented from four given descriptors, which were presented in writing and in pictures. In addition the descriptors were read to the children. The children were allowed to smell each odor as often as necessary but had to choose one of the four given descriptors (4 alternative forced choice). The sum of the correct answers was regarded as the odor identification score.

### Part two

Two odors were excluded from the original odor identification test according to the results of part one. Thus resulting in the 14-item odor identification test (“Sniffin' Kids” test). Therefore the data analysis of the 537 children was repeated to obtain normative data for the 14-item odor identification test. The body mass index (BMI-Z-scores) was recorded to observe the influence of the BMI on the odor identification results in 81 children (45 girls, 36 boys).

#### Test validity

The validity of the test, to distinguish between normosmia and anosmia in children, was investigated by comparing odor identification scores of children with isolated congenital anosmia (ICA) to the healthy control group (n = 537).

#### Anosmic children

Twenty-five children with ICA were included who were tested in our Smell & Taste Clinic between 2005 and 2010 with a mean age of 12 years (SD 2.7, range 8–17 years), (Table 1). All ICA subjects were referred to the Department of Otorhinolaryngology at the TU Dresden by other Departments of this University (e.g., Pediatrics and Neurology) or they presented themselves to the smell dysfunction clinic of the Department of Otorhinolaryngology. All subjects were in good health with no signs or symptoms except for anosmia. Upon careful questioning none of these patients could remember any odorous sensations apart from intranasal sensations likely to be mediated by the trigeminal nerves. All of the ICA subjects had MRI scans of the brain; none of them had any major cranial malformation as verified by T1- and T2-weighted MRI sequences. In addition to psychophysical testing most ICA subjects – whenever deemed necessary - also received electrophysiological testing using chemosensory event-related potentials; none of the tested ICA patients had electrophysiological responses to olfactory stimuli.

#### Test reliability

To test the reliability of the 14-item odor identification test, a subgroup of 31 children (19 girls, 12 boys; mean age 11.7 years, SD 1.33 years) was tested a second time 4-6 months after the first session.

### Statistical analyses

Descriptive statistics were obtained for the odor identification scores. In addition the percentage of correct identification for each individual odor was calculated. The data was analyzed by means of SPSS 22.0 (SPSS Inc., Chicago, IL, USA). T-tests were used whenever appropriate. The data of the 16- as well as the 14-item odor identification test was not normally distributed as evaluated by the Kolmogorow-Smirnow-Test (p<0.0.001 for both data sets). Therefore non-parametric tests (Cochran, Wilcoxon-Test, Mann-Whitney-U-Test and Kruskal-Walis-Test) were used whenever appropriate. In addition Spearman's correlations were used. The level of significance was set at 0.05. Degrees of freedom are written in subscript when indicated.

## Results

### Part one

In this study 537 children (268 girls, 269 boys) with an age range of 6-17 years (mean 11.9 years, SD 3.10 years) were included. The age distribution between girls and boys was not significantly different (t_535_ = 0.19, p = 0.85).

**Figure 1:**
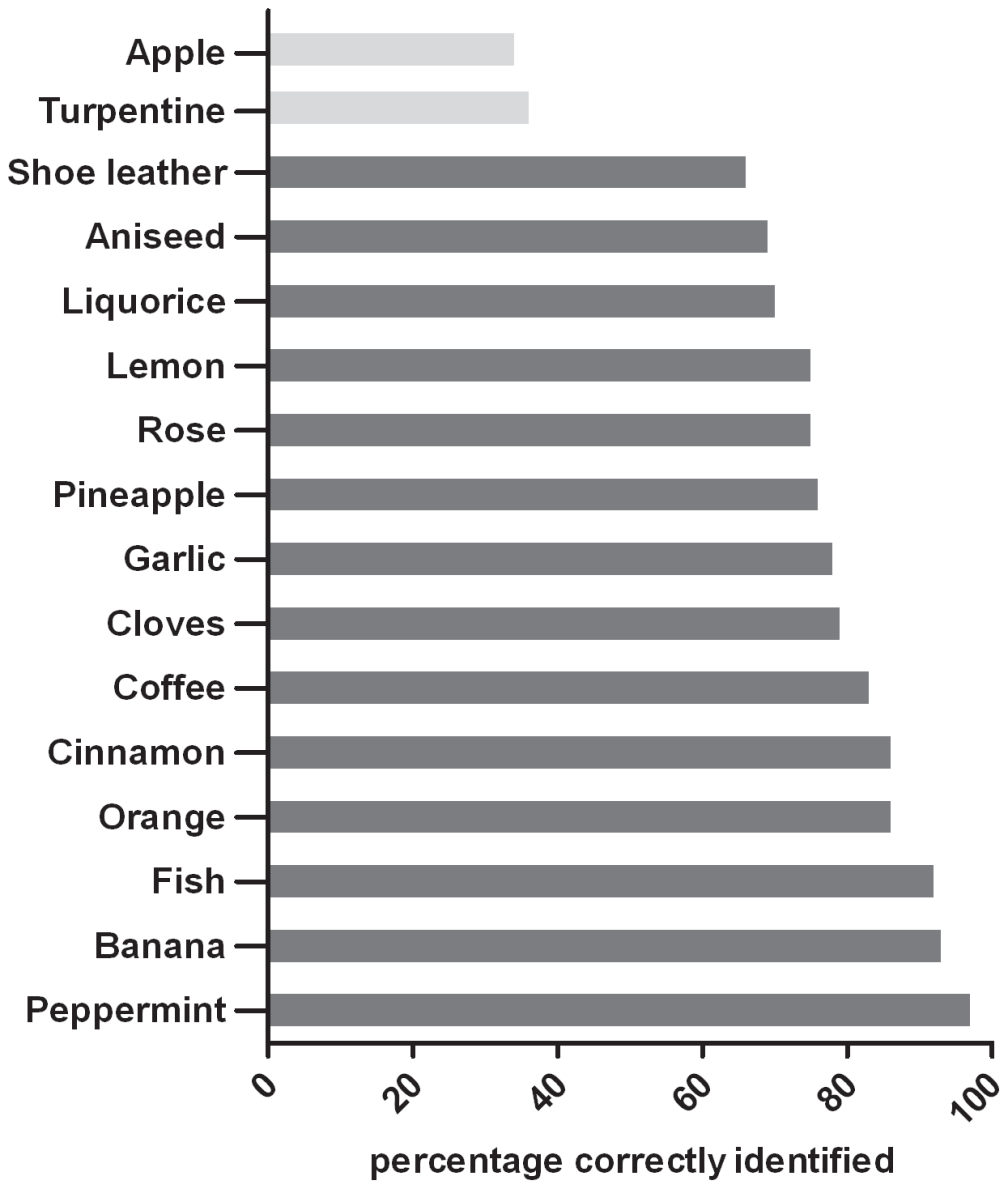
Percentage of correctly identified odors. Displayed are the percentages of correct identification of the 16-item odor identification test for all children (n = 537). The odors, which were chosen for the 14-item “Sniffin' Kids” test are marked in dark grey. Odors, which were excluded were significantly less often correctly identified and are displayed in light grey.

Children performed with a mean of 11.98 points (SD 2.07, range 2-16 points) on the 16-item odor identification test. The percentage of correct identification for each item was calculated to identify odors, which are not familiar to children. Listed from high to low mean percentage of correct identification: Peppermint: 97%, Banana: 93%, Fish: 92%, Orange: 86%, Cinnamon: 86%, Coffee: 83%, Cloves: 79%, Garlic: 78%, Pineapple: 76%, Rose 75%, Lemon: 75%, Liquorice: 70%, Aniseed: 69%, Shoe leather: 66%, Turpentine: 36%, Apple: 34% (Figure 1). A Cochran-test revealed significant differences between the identification of the 16 odors (Q_15_ = 127.62, p<0.001). Multiple Bonferroni adjusted pairwise comparisons showed that the odors Apple and Turpentine were significantly less often correctly identified compared to all other odors (U between 8.56–17.77, all ps<0.001). Because of that, the items Apple and Turpentine were excluded, forming a 14-item odor identification test, the “Sniffin' Kids” test (Figure 1).

### Part two

**Figure 2:**
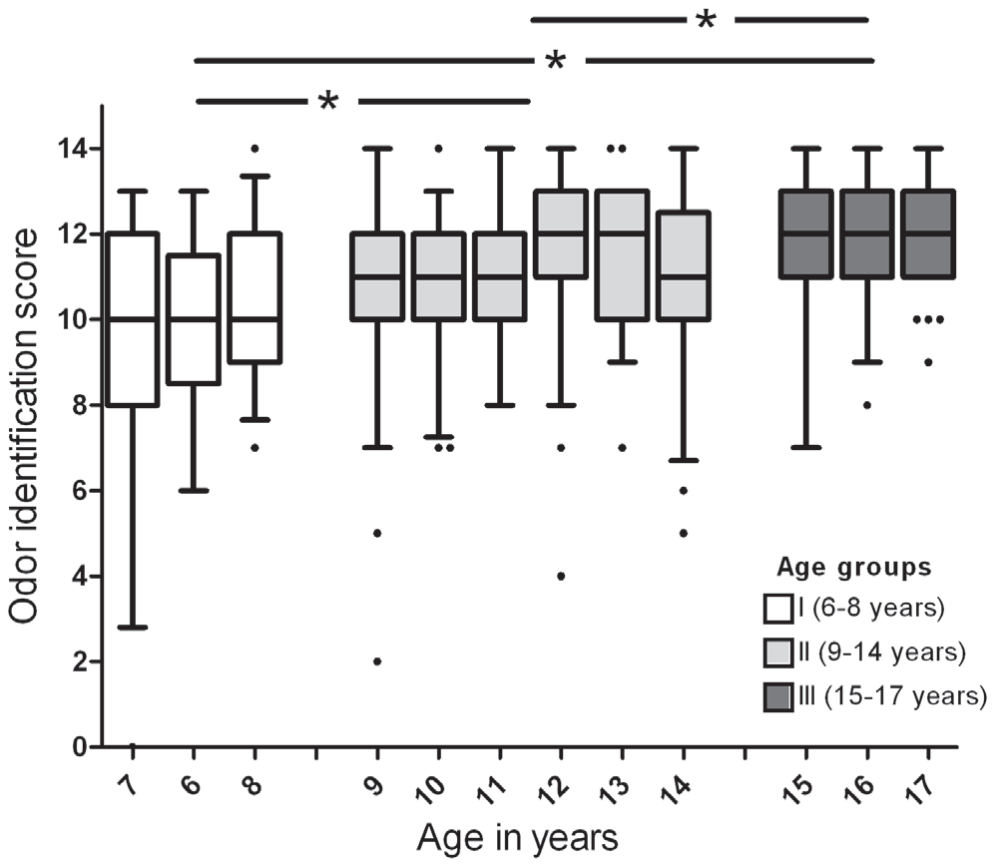
Odor identification score and age groups. The boxplot displays the mean odor identification score for all ages. The age groups I, II and III differ significantly by means of odor identification score, while no age difference was obtained within a group. (* = p<0.001).

The odors chosen for the “Sniffin' Kids” test are listed in Table 2. For all participants the mean odor identification score was 11.22 (SD 1.87, range 2–14). A detailed description of odor identification for each individual item can be found in Table 3. No sex difference (U_535_ = 1.29, p = 0.20) but a positive correlation between odor identification score and age was observed (ρ_537_ = 0.29, p<0.001). In addition to this correlation the age of children had a significant effect on odor identification performance (*Χ* ^2^ = 59.26, p<0.001) with older children reaching higher scores. Therefore we divided the sample into subgroups: group I (6–8 years), group II (9–14 years) and group III (15–17 years). A significant difference in odor identification performance was found between groups (*Χ* ^2^ = 51.37, p<0.001) (Figure 2), with mean sores increasing from group I to group III. Within each group the age did not affect the odor identification score (Group I: *Χ* ^2^ = 0.59, p = 0.74; II: *Χ* ^2^ = 7.28, p = 0.20; III: *Χ* ^2^ = 0.23, p = 0.89). In line with this, no correlation between age and odor identification score was found within the age groups (Group I: ρ_76_ = 0.09, p = 0.45; II: ρ_344_ = 0.06, p = 0.28; III: ρ_117_ = 0.01, p = 0.93).

**Table 2:** Items of the “Sniffin' Kids” test.

“Sniffin' Sticks” number	Odor	Descriptor 2	Descriptor 3	Descriptor 4
1	**Orange**	Blackberry	Strawberry	Pineapple
2	**Leather**	Smoke	Glue	Grass
3	**Cinnamon**	Honey	Vanilla	Chocolate
4	**Peppermint**	Chives	Wood	Onion
5	**Banana**	Coconut	Walnut	Cherry
6	**Lemon**	Peach	Apple	Grapefruit
7	**Liquorice**	Gummibears	Chewing gum	Cookies
9	**Garlic**	Onion	Sauerkraut	Carrot
10	**Coffee**	Cigarette	Wine	Candle smoke
12	**Cloves**	Pepper	Cinnamon	Mustard
13	**Pineapple**	Pear	Plum	Peach
14	**Rose**	Chamomile	Raspberry	Cherry
15	**Aniseed**	Rum	Honey	Wood
16	**Fish**	Bread	Cheese	Ham

The Table shows the 14 odors and their descriptors, which were selected for the “Sniffin' Kids” test from the “Sniffin' Sticks” 16-item odor identification test. The number is accordant to the 16-item odor identification test.

**Table 3:** Results of the odor identification test.

		Control				
Odor		All ages	Group I (6–8)	Group II (9–14)	Group III (15–17)	Anosmic
**Orange:**	All	86 (83–89)	81 (73–91)	85 (81–88)	93 (89–98)	36 (16–56)
	Girls	88 (83–91)	89 (79–98)	85 (79–91)	94 (88–100)	39 (14–64)
	Boys	84 (80–88)	72 (55–88)	84 (79–90)	92 (85–100)	29 (0–74)
**Leather**	All	66 (62–70)	54 (43–65)	67 (62–72)	74 (65–82)	12 (0–26)
	Girls	67 (61–72)	48 (32–63)	67 (60–75)	77 (67–88)	17 (0–36)
	Boys	66 (60–72)	63 (45–80)	66 (59–73)	69 (55–82)	0 (0)
**Cinnamon**	All	86 (82–88)	91 (84–97)	86 (82–90)	81 (74–88)	28 (9–47)
	Girls	87 (82–90)	93 (85–100)	88 (83–93)	76 (69–89)	28 (5–51)
	Boys	85 (80–89)	88 (75–100)	84 (79–90)	84 (74–95)	27 (0–74)
**Peppermint**	All	97 (95–98)	91 (84–97)	97 (96–99)	99 (98–100)	48 (27–69)
	Girls	97 (94–99)	91 (82–100)	98 (96–100)	100 (100)	44 (19–70)
	Boys	96 (94–99)	91 (80–100)	97 (94–99)	98 (94–100)	57 (8–100)
**Banana**	All	93 (90–95)	84 (76–93)	93 (90–96)	97 (95–100)	20 (3–37)
	Girls	95 (90–96)	86 (76–97)	95 (92–98)	97 (93–100)	17 (0–36)
	Boys	91 (88–95)	81 (67–96)	91 (87–96)	98 (94–100)	29 (0–74)
**Lemon**	All	75 (70–78)	76 (67–86)	72 (67–77)	81 (74–88)	36 (16–56)
	Girls	71 (65–76)	77 (64–90)	65 (58–73)	80 (71–90)	28 (5–51)
	Boys	78 (74–83)	75 (60–91)	78 (72–84)	82 (72–93)	57 (8–100)
**Liquorice**	All	70 (66–74)	55 (44–67)	72 (67–77)	74 (65–82)	48 (27–69)
	Girls	70 (64–75)	54 (39–70)	74 (67–81)	71 (60–82)	44 (19–70)
	Boys	70 (64–75)	56 (38–74)	70 (64–77)	77 (64–89)	57 (8–100)
**Garlic**	All	78 (74–81)	55 (44–67)	80 (76–84)	86 (80–83)	44 (23–65)
	Girls	77 (71–82)	59 (44–74)	78 (71–84)	86 (78–95)	39 (14–64)
	Boys	78 (74–83)	50 (31–68)	81 (76–87)	86 (77–96)	57 (8–100)
**Coffee**	All	83 (79–85)	84 (76–93)	81 (77–85)	86 (80–93)	16 (0–31)
	Girls	81 (76–86)	80 (67–92)	79 (73–86)	88 (80–96)	6 (0–17)
	Boys	84 (79–88)	91 (80–100)	82 (77–88)	84 (74–95)	43 (0–92)
**Cloves**	All	79 (75–82)	65 (54–76)	81 (76–85)	82 (75–89)	16 (0–31)
	Girls	82 (76–86)	73 (59–86)	82 (76–88)	86 (78–95)	17 (0–36)
	Boys	76 (70–81)	53 (34–71)	79 (73–85)	77 (64–89)	14 (0–49)
**Pineapple**	All	76 (72–80)	68 (58–79)	74 (70–79)	87 (81–93)	28 (9–47)
	Girls	78 (72–82)	64 (48–78)	77 (70–83)	89 (82–97)	17 (0–36)
	Boys	75 (70–81)	75 (60–91)	72 (66–79)	84 (74–95)	57 (8–100)
**Rose**	All	75 (71–78)	63 (52–74)	74 (70–79)	84 (77–91)	32 (12–52)
	Girls	79 (74–84)	66 (51–81)	80 (73–86)	86 (78–95)	44 (19–70)
	Boys	71 (65–76)	60 41–77)	70 (63–77)	80 (69–92)	0 (0)
**Aniseed**	All	69 (64–72)	58 (47–69)	66 (61–71)	83 (76–90)	20 (3–37)
	Girls	69 (63–74)	61 (46–76)	66 (58-73)	82 (72–91)	28 (5–51)
	Boys	68 (62–74)	53 (35–71)	66 (59–73)	84 (74–95)	0 (0)
**Fish**	All	92 (89–94)	83 (74–92)	92 (89–95)	97 (95–100)	28 (9–47)
	Girls	91 (87–94)	86 (76–97)	91 (86–95)	97 (93–100)	33 (9–58)
	Boys	92 (89–95)	78 63–93)	93 (89–97)	98 (94–100)	14 (0–49)

Displayed are the mean percentage of correct identification for each odor for the control and anosmic children. The percentages are shown for all, girls, boys and each age group separately. In addition the 95% confidence interval is shown in brackets.

The three groups scored on the 14-item odor identification test as followed: Group I (n = 76): mean odor identification score 10.09 points (SD 1.98, range 4–14 points). Group II (n = 344): mean odor identification score 11.19 points (SD 1.87, range 2–14 points). Group III (n = 117): mean odor identification score 12.05 points (SD 1.33, range 7–14 points). No sex differences were found in odor identification scores in all three groups (Table 4).

**Table 4:** Test results for different age groups.

Group	Participants	Girls/Boys	Age	Identification score	T-test, girls/boys	Kruskal-Walis-Test effect of age
**I (6**–**8)**	76	44/32	7.3 (0.7)	10.09 (1.98)	t = 0.93, p = 0.36	*Χ* ^2^ = 0.56, p = 0.74
**II (9**–**14)**	344	158/186	11.4 (1.8)	11.19 (1.87)	t = 0.50, p = 0.62	*Χ* ^2^ = 7.28, p = 0.20
**III (15**–**17)**	117	66/51	16.3 (0.7)	12.05 (1.33)	t = 0.79, p = 0.43	*Χ* ^2^ = 0.23, p = 0.89

Odor identification scores are shown for the three age groups in addition to descriptive data of the age groups. Displayed are mean (SD). No sex difference was found between girls and boys on the odor identification test for all three age groups. In addition no effect of age was observed within an age group.

To separate normosmia from olfactory dysfunction with the “Sniffin' Sticks” test the 10^th^ percentile was used [3]. We applied this cutoff to our data sample. According to the 10^th^ percentile a score of >7 in age group I, a score of >8 in age group II and a score of > 10 in age group III is considered normosmic. Therefore scores below these values can be considered as hyposmic. According to this definition, 6 (7.9%) children in group I, 29 (8.4%) children in group II and 22 (11.1%) children in group III had olfactory dysfunction.

To evaluate the reliability of the olfactory test, a group of 31 children from age group II (mean age 11.7, SD 1.3 years, 12 girls, 19 boys) was tested again after a mean interval of 4–6 months. The mean odor identification score for the first testing was 11.58 points (SD 1.61) and for the second testing 12.23 points (SD 1.23). A test-retest reliability of ρ = 0.44 (p = 0.012) was observed.

**Figure 3:**
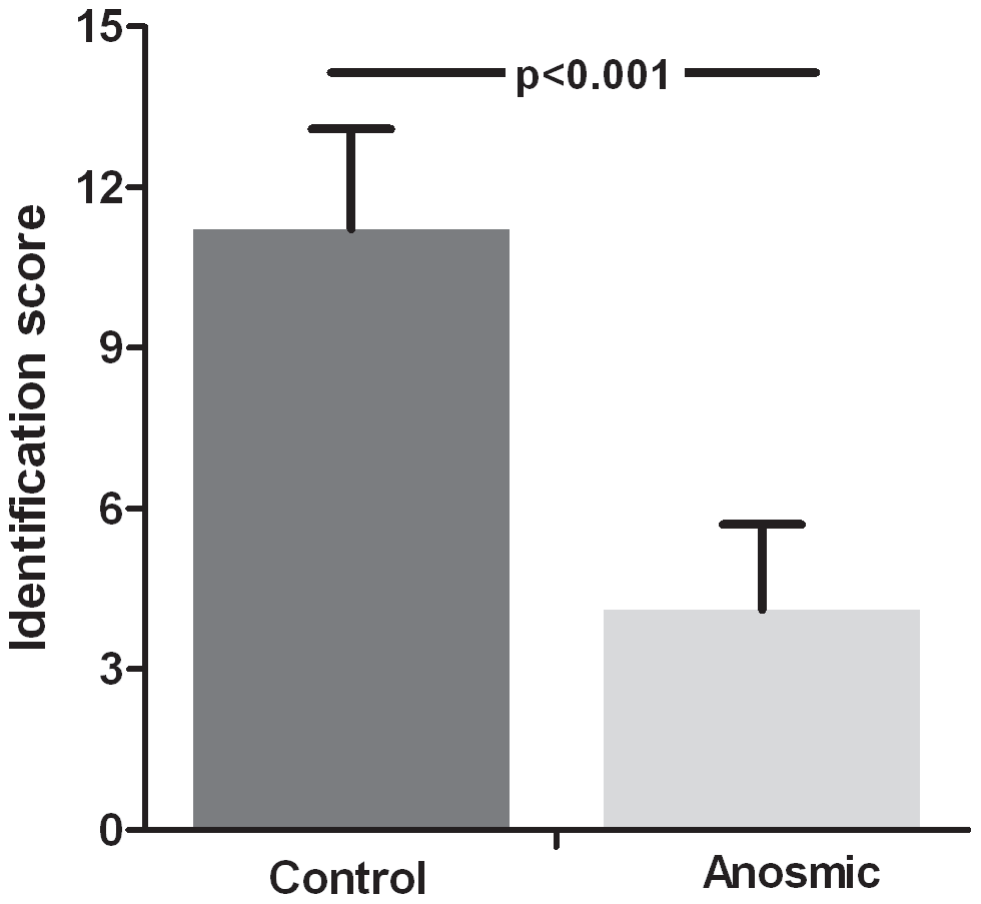
Comparing anosmic and healthy children. Odor identification scores of healthy children, dark grey, (n = 537) and congenital anosmic children, light grey, (n = 25) are shown (means, one standard deviation). Healthy children scored in average 11.22 (1.87) points on the “Sniffin' Kids” test, while congenital anosmic children scored 4.12 (1.59) points. This was significantly different with a p value <0.001.

For validation of the 14-item odor identification test, results from a group of congenitally anosmic children (18 girls, 7 boys, no age difference to healthy group (t_560_ = 0.49, p = 0.63)) were compared to the above-mentioned results. Children in the anosmic group scored on average 4.12 points (SD 1.59; range 2–7 points) on the odor identification test. When compared to the group of healthy children (n = 537) a significant difference in olfactory performance was observed (U_560_ = 8.46; p<0.001) (Figure 3). The group of anosmic children was too small to meaningfully divide it into the three age groups used above; only 2 children would be in group I, 16 in group II and 7 in group III. None the less all children in the anosmic group scored below 8 points, which is considered to indicate a reduced sense of smell in all three age groups.

Possible effects of BMI on the odor identification score were observed in a subgroup of healthy children (n = 81). For this the BMI-Z-scores were calculated. An average of 12.0 points (1.56, range 7–14 points) was achieved on the odor identification test. No correlation between BMI-Z-scores and odor identification was found (ρ = 0.06, p = 0.62).

## Discussion

In the current study we evaluated the “Sniffin' Sticks” 16-item odor identification test in a large population of children. Results from part one shows that some odors of the test, especially Apple and Turpentine, are not familiar to children. Thus it was necessary to modify this test to be more suitable for children.

Although olfactory impairment is less common in children than in adults [22], recent studies have shown a reduced sense of smell in children due to several reasons like head trauma [11], [23], [24], adenoid hypertrophy [25], anorexia nervosa [26] or other psychiatric diseases [27]. Therefore there is need for a reliable, valid and easy to use test for measuring olfactory function in children [28]. To date there are a few odor identification tests, which have been developed for children. The “Smell Wheel” and the odor identification test of the NIH Toolbox were developed for the USA. Children from Europe are not familiar with odors such as Play-Doh, which could lead to lower odor identification scores and/or increased variance when using these tests. Laing et al. developed an odor identification test in Australia for children aged 5-7 years using squeeze bottles for odor presentation [15]. In Europe a self-developed odor identification test was introduced to be used in a Polish population [18]. The first two tests are not commonly used in Europe and the later tests are not commercially available. The shorter 12-item “Sniffin' Sticks” odor identification test was evaluated in children in a Dutch population [8]. In our study we used the same odor presentation method – the “Sniffin' Sticks”. The benefit of our current study is the odor selection and therefore choosing odors, which are well identified by children. The odors were selected from the original “Sniffin' Sticks” 16-item odor identification test resulting in a 14-item test. In addition two odors, Aniseed and Garlic, which are not included in the 12 odors tested in the Dutch population, have shown to be well known by children.

In the current study, a large group of 537 children between 6-17 years of age were included. We did not include children younger than six years, because previous studies have shown that odor identification is difficult and not reliable in children less than six years of age [20], [29]. In contrast to that, children starting from age 3 years were included in one study [18]. Results from these children might be biased, because, if unknown, parents were allowed to explain the descriptors to the children. In our study all children understood the task and were able to perform the test. Due to unfamiliar items the odor identification test was modified excluding Apple and Turpentine. All other odors were identified at rates between 66-97%. Previous studies reported the validity of 12-item odor identification tests [30], [31]. In an olfactory screening test a subset of odors were taken from the original “Sniffin' Sticks” test [31]. The 12-item cross-cultural smell identification test (CC-SIT) is a derivative of the UPSIT 40-item odor identification test [2], [30]. Both tests have proven to be useful especially in a clinical setting. Thus, it is plausible that a 14-item odor identification test exhibits similar qualities. Whether women outperform men in odor identification tests has been controversially debated [3], [8]. In line with previous studies no sex difference was found in the current study in odor identification scores [3], [16], [18], [20]. It has been described that odor identification improves with age in children [14], [16], [20]. This was also the case in the current study. Therefore we created three age groups, which differed significantly in odor identification scores from one another. Within each group the odor identification score was not affected by age. For presenting normative values of an odor identification test it is necessary to obtain stable results within a population. This was achieved by forming the three age groups. This allowed us to establish normative data for each age group with a cut off value at the 10^th^ percentile of odor identification scores, which separates normosmia from impaired olfactory function. The 10^th^ percentile is an established value for separating normosmia from hyposmia in adults when using the “Sniffin' Sticks” test battery [3] or when using the UPSIT [2]. We were able to show that the “Sniffin' Kids” test is able to discriminate between normosmia and impaired olfactory function by including congenital anosmic children in the study. Interestingly, none of the above mentioned odor identification tests for children have been validated this way [8], [14]–[18]. In our study all congenital ansomic children scored below the 10^th^ percentile on the “Sniffin' Kids” test. In addition to this validation, the “Sniffin' Kids” test showed to be reliable for the tested age group with a reliability value of ρ = 0.44. This value is smaller when compared to the test-retest reliability of the “Sniffin' Sticks” 16-item odor identification test in adults [21]. It has to be considered that the population tested in this study was fairly small (n = 31) and that the number of items was reduced to 14. In addition, the small coefficient of correlation is also explained by the relative homogeneity of the group tested because no children with diminished or absent olfactory function had been included here. Since the reliability was only tested in children from age group II (9-14 years) further studies are needed to evaluate the reliability especially in age group I (6–8 years). The interval between the first and second testing was between 4 to 6 months. The exact dates of testing were not available. Therefore it was not possible to study any effects of interval lengths on the outcome of the reliability. In line with previous findings the BMI (BMI-Z-score) of children had no effect on the odor identification performance [8]. It has to be considered that in our study only four children had a BMI-Z-score of ±2 from the mean. This is in contrast to a study reporting changed odor identification abilities in dependence of the BMI in children [9].

The “Sniffin' Kids” test is based on the original “Sniffin' Sticks” 16-item odor identification test, which is largely used in clinics and laboratories throughout Europe. We modified this test rather than creating a new test from scratch. Therefore it is possible to test children as well as adults with portions of the same test battery. This is considered an advantage compared to the “Smell Wheel” and the NIH Toolbox, which are not reusable and for the “Smell Wheel” not applicable in adults, making these tests much more costly than the “Sniffin' Kids” test.

The sample size of our study was fairly large to obtain normative data for the “Sniffin' Kids” test. Nonetheless further studies have to be conducted to strengthen these results, especially in the age group from 6-8 years of age. The original 16-item odor identification test was developed in Germany and evaluated in a number of other European countries. Children included in the study grew up in Germany. Both the NIH-toolbox and the “smell wheel” were administered to children, who grew up in the USA [14], [16]. Additional studies are necessary to evaluate the “Sniffin' Kids” test in other countries especially countries outside of Europe.

Further studies are needed to evaluate possible influences of oral and nasal surgery on the outcome of odor identification score as has been shown previously [8]. A possible shortcoming of the study is that no cognitive test was conducted. Therefore the influence of cognition on odor identification ability could not been observed in the current study.

## Conclusion

With the 14-item odor identification test, the “Sniffin' Kids” test, we propose a valid and reliable method for olfactory testing in children between 6–17 years of age. We provide normative data for three age groups from a large sample size.
